# How to Maximally Support Local and Regional Biodiversity in Applied Conservation? Insights from Pond Management

**DOI:** 10.1371/journal.pone.0072538

**Published:** 2013-08-12

**Authors:** Pieter Lemmens, Joachim Mergeay, Tom De Bie, Jeroen Van Wichelen, Luc De Meester, Steven A. J. Declerck

**Affiliations:** 1 Laboratory of Aquatic Ecology, Evolution and Conservation, Leuven, Belgium; 2 Research Institute for Nature and Forest, Kingdom of Belgium, Geraardsbergen, Belgium; 3 Laboratory of Protistology and Aquatic Ecology, Ghent University, Ghent, Belgium; 4 Department of Aquatic Ecology, Netherlands Institute of Ecology (NIOO-KNAW), Wageningen, The Netherlands; Consiglio Nazionale delle Ricerche (CNR), Italy

## Abstract

Biodiversity and nature values in anthropogenic landscapes often depend on land use practices and management. Evaluations of the association between management and biodiversity remain, however, comparatively scarce, especially in aquatic systems. Furthermore, studies also tend to focus on a limited set of organism groups at the local scale, whereas a multi-group approach at the landscape scale is to be preferred. This study aims to investigate the effect of pond management on the diversity of multiple aquatic organism groups (e.g. phytoplankton, zooplankton, several groups of macro-invertebrates, submerged and emergent macrophytes) at local and regional spatial scales. For this purpose, we performed a field study of 39 shallow man-made ponds representing five different management types. Our results indicate that fish stock management and periodic pond drainage are crucial drivers of pond biodiversity. Furthermore, this study provides insight in how the management of eutrophied ponds can contribute to aquatic biodiversity. A combination of regular draining of ponds with efforts to keep ponds free of fish seems to be highly beneficial for the biodiversity of many groups of aquatic organisms at local and regional scales. Regular draining combined with a stocking of fish at low biomass is also preferable to infrequent draining and lack of fish stock control. These insights are essential for the development of conservation programs that aim long-term maintenance of regional biodiversity in pond areas across Europe.

## Introduction

In an effort to restore and maintain biodiversity, the European Union has directed efforts towards the conservation of habitats and species through the designation of protected areas [1]. Many of these habitats are located in heterogeneous anthropogenic landscapes [2,3] and the intended nature values often result from traditional land-use practices [4]. During the last decades, many of these practices have been abandoned or have been strongly modified as the result of a variety of socio-economic developments [5]. Current European biodiversity conservation programs in anthropogenic landscapes therefore often involve the maintenance or imitation of traditional land use practices [6]. Most of these programs are directed towards terrestrial landscapes, but also in standing waters traditional management practices have been lost, and restoring these may potentially contribute to local and regional biodiversity.

Although important efforts have been made for the conservation of semi-natural habitats, research on the impact of specific management practices on biodiversity is remarkably sparse [7,8] and typically focuses on one or a very limited number of taxonomic groups [9], such as birds [10,11] or butterflies [12]. This is surprising since the focus of conservation efforts tends to increasingly shift from single to multispecies approaches [13,14]. Indeed, low congruency among the diversity of multiple organism groups has often been observed [15–17] and the use of indicator taxa is increasingly being questioned [18–20]. Conservation schemes should generally aim at maintaining biodiversity of a broad variety of organism groups. Furthermore, while most studies are geared at evaluating restoration measures at relatively small, local scales, conservation schemes should take a regional perspective and try to ensure the long-term maintenance of biodiversity at the landscape scale. Such aims require a profound knowledge of how different groups of organisms respond to different management regimes and which combinations of these regimes may ensure a maximization of biodiversity at larger spatial scales.

Several studies have highlighted the importance of ponds for aquatic biodiversity at regional scales [21–24]. Ponds often contain rare, protected and endemic species [25,26]. In Western Europe ponds are often of anthropogenic origin [27] and traditional management practices, such as low-intensity fish farming, often allowed the persistence of high nature values, such as high levels of biodiversity and the occurrence of rare and endangered species. However, eutrophication and pollution combined with the abandonment or modification of management practices has resulted in a considerable decrease in habitat quality and the loss of species [28,29]. Although important efforts to restore and maintain aquatic biodiversity have been made, evaluations on management measures have mainly focused on their effects on the physical and chemical environment or on food web structure and dynamics [30–32]. Studies on the impact of management practices on diversity at local and regional spatial scales remain scarce (but see 33).

With this study, we aimed to investigate the impact of different management practices on biodiversity in ponds. We evaluate their effect on the diversity of multiple taxonomic groups (e.g. phytoplankton, zooplankton, several groups of macro-invertebrates, submerged and emergent macrophytes) simultaneously. To accomplish this, we surveyed a total of 39 shallow ponds, representing five major pond management types differing in fish stock management and in the frequency of pond drainage. Rather than focusing solely on the effects of management at the level of individual ponds, we also took a broader approach by evaluating the relative contribution of different management types to regional biodiversity in the studied pond network, with special attention for the occurrence of rare and endangered species.

## Methods

### 1: Ethics statement

We sampled in accordance to the European directive 2010/63/EU and had explicit permission of respective owners (fish farmers and the Agency for Nature and Forests) to enter private property. No additional permissions were required for this study.

### 2: Study area

**Figure 1:**
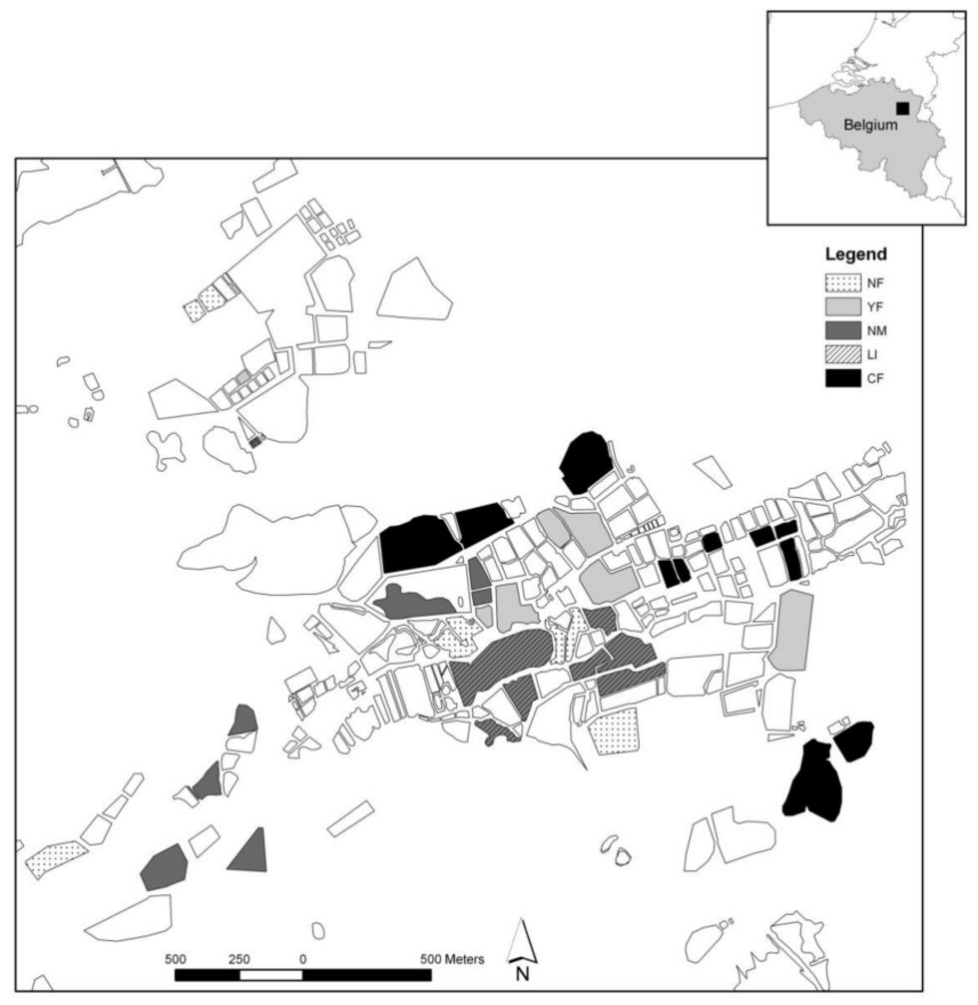
Overview of a part of "Vijvergebied Midden-Limburg" with the selected ponds representing the different management types. See Table 1 for a detailed description of each pond management type. Note that one NF-pond, situated approximately 2 kilometers east of the depicted ponds, is not drawn on the map.

This study was performed in the region called “Vijvergebied Midden-Limburg”, which is situated in the North-eastern part of Belgium (50°59'00.92″ N; 5°19'55.85″ O and surroundings) (Figure 1) and is part of "De Wijers" area. The region comprises more than 1000 shallow ponds [34], many of which originated from the extraction of iron ore (between 1850 and 1900) and peat (until 1930) [35]. For the purpose of fish farming, additional ponds were created after 1950. The main water sources are two streamlets (Oude and Nieuwe Roosterbeek). Ponds are connected to each other by a complex network of rivulets. Fish farming, still an important local practice, has strongly intensified during the last three decades. In ponds used for intensive fish culture, the use of artificial feeds (up to 1400 kg ha^-1^) and fertilizers have resulted in a strong increase in fish biomass (up to 1200 kg ha^-1^) and habitat degradation, such as the disappearance of submerged macrophytes and the frequent occurrence of cyanobacteria blooms. Yet the pond complex is still highly reputed for its biological diversity and it is currently protected by several national and international legislations (Natura 2000 status, Birds directive [79/409/EEC] and Habitats directive [92/43/ECC]). Recently, a considerable number of ponds were acquired by the Flemish government (Agency for Nature and Forests) and are now managed for purposes of nature conservation.

### 3: Pond selection and data collection

We identified five major pond management practices in the area (Table 1). Two of these practices target nature conservation goals and are applied by the governmental agency responsible for the area (Agency for Nature and Forests, ANB): a small number of ponds are managed with the intention to keep them fishless (NF), while a larger set of ponds are exposed to irregular and low-intensity management (LI), the latter mainly involving occasional dry-stands (approximately every five years, but irregularly spaced in time). Two practices are commercial and performed by local fish farmers. The most common of these practices is the intensive farming of common carp *Cyprinus* *carpio* (L.) (further referred to as carp ponds, CF). Some ponds are also used for raising juvenile cyprinids, such as common carp and ide *Leuciscus* *idus* (L.) (YF). Finally, a large proportion of the ponds in the area remains unmanaged for long periods of time (NM). The management practices differ not only in fish stock management, but also in the frequency of drainage and in measures to prevent fish immigration and emigration. Carp farming ponds (CF), fishless ponds (NF) and ponds with young-of-the-year fish (YF) are drained annually during winter. Carp farming ponds are initially stocked with relatively high biomasses of common carp, often accompanied by other species, such as tench *Tinca* *tinca* (L.). Initial stocking biomasses are approximately 100 kg ha^-1^. The management of NF-ponds is entirely focused on preventing the establishment of fish populations, whereas YF-ponds are initially stocked with low densities of young-of-the-year fish. These ponds are only stocked with fish around May, allowing the development of lush emergent and submersed vegetation in the months prior to stocking. During the filling of NF- and YF-ponds, the inlets are covered with stainless steel grids (mesh size, 2mm) to prevent immigration of fish from outside the pond. Ponds with low intensity management (LI) are only occasionally drained (every three to six years); the last time these ponds were drained prior to the present study varied from two to three years. After drainage, LI-ponds were stocked with intermediate densities of pike *Esox* *lucius* (L.), roach *Rutilus* *rutilus* (L.), rudd *Scardinius* *erythrophthalmus* (L.) and tench (total fish biomass, 40 kg ha^-1^), whose population development was subsequently not controlled. Ponds without management (NM) were never stocked and are seldom or never drained. They haven’t been drained in more than 10 years prior to our study. For our survey, we randomly selected replicate ponds for each management type (n = 7, except for CF where n = 11) (Figure 1). The ponds were surveyed either in 2006 or 2007 (2006, n = 22; 2007, n = 17), making sure that all management types were more or less equally represented in both years (2006: NF, n = 3; YF, n = 4; NM, n = 4; LI, n = 4; CF, n = 8 and 2007: NF, n = 4; YF, n = 3; NM, n = 3; LI, n = 3; CF, n = 3).

**Table 1:** Description of the pond management types in the order of increasing fish biomass.

Pond Management Type	Main Purpose	Pond Drainage	Fish Stock Management
No fish (NF)	To create fishless ponds as nature conservation measure (mainly for amphibians).	Ponds are drained annually in autumn and refilled in early spring.	There is no stocking of fish and nets are placed on the inlets to prevent immigration of fish.
Commercial farming of young of the year fish (YF)	The extensive rearing of young-of-the-year-fish (typically common carp and ide).	Ponds are drained in autumn and refilled in spring.	Nets are placed on the inlets to prevent immigration of wild fish. The ponds are stocked with fish fry in late spring when vegetation has already developed. Fish is harvested in autumn.
No management (NM)	No specific purpose.	No drainage for more than ten years.	No fish stock management. Fish can freely move in and out the ponds via rivulets.
Low intensive management (LI)	To create ponds with indigenous fish communities for nature conservation purposes.	Ponds are occasional drained (approximately every five years, but irregularly spaced in time).	Two or three years prior to this study, ponds were drained, refilled and initially stocked with adult rudd, tench and pike (40 kg ha^-1^). Fish can freely move in and out the ponds.
Carp farming (CF)	Commercial semi-intensive farming (mainly common carp, 1000 kg ha^-1^ year^-1^).	Annual or bi-annual winter drainage to harvest fish.	Ponds are stocked with 100 kg ha^-1^ of fish in spring. Ponds are partly or completely covered with wire netting to minimize predation by piscivorous birds. Use of artificial feeds (ca. 1400 kg ha^-1^ year^-1^) to increase fish production.

Pond surfaces were calculated with the GIS software package ArcView GIS 3.2a (ESRI, Inc.). We measured maximum pond depths once during summer with a graduated stick at the deepest point of each pond (nearby the outlet). At the same moment, we estimated the thickness of the silt layer from the profile of sediment cores taken at 2 random chosen spots in the deeper part of the ponds. We used electrodes (WTW multiline F meter, Geotech ©) to measure pH and daytime oxygen concentration in spring (May) and summer (July). Water transparency was determined during spring and summer campaigns using a Snell tube [36]. Using a tube-sampler (length 1.2 m; diameter 75 mm), we took depth-integrated samples in the open water at five locations in each pond during spring (May) and summer (July). The samples were pooled and subsamples of 1 L were immediately stored on ice in the dark for further analysis of suspended solids, chlorophyll *a* and nutrient concentrations. Suspended solids were determined gravimetrically in the laboratory by filtering a known volume of pond water on pre-weighed GF/F filters (Whatmann). Chlorophyll a concentrations were measured spectrophotometrically according to Ritchie [37] after methanol extraction [38]. We measured total concentrations of nitrogen (TN) and phosphorus (TP) after alkaline persulfate digestion [39] on a Technicon Autoanalyzer II (Technicon, Tarrytown, New York, USA).

Fish community characteristics were determined by placing multiple (n =3-5, dependent on pond surface area) double fyke nets (length 7.7 m, mesh size 8 mm) in each pond for 24 hours. Specimens were identified, measured (fork length) and weighted. The total biomass of each species per pond was expressed as catch per unit effort (CPUE; kg per fyke net).

Zooplankton and phytoplankton communities were sampled quantitatively during summer in the littoral and pelagic zone of each pond. Using a tube sampler, we collected depth-integrated samples (25 L) at five randomly chosen locations in both mesohabitats separately. At very shallow locations we used a 5 L-beaker. Samples from both habitats were pooled together. For zooplankton samples, we filtered 40 L of this combined sample through a conical plankton net (mesh size, 64 µm). We used 250 mL from the pooled sample to characterize the phytoplankton community. Zooplankton and phytoplankton samples were preserved in formaldehyde (4%).

Cladocerans were identified to species level using Flössner [40] and counted. *Daphnia* *galeata* (Sars) and *D* *. longispina* (Müller) were considered as one taxon. Copepods were divided in two main groups (Cyclopoids and Calanoids) and counted. Taxon richness in zooplankton was estimated through rarefaction, with cut off values at 300 individuals using the software Primer v5 [41]. For the analysis of phytoplankton taxon richness, we identified 200 cells, coenobia or colonies from each sample to genus level using John et al. [42].

Aquatic macro-invertebrates were sampled twice a year (May and July) by intensive sweeping in the littoral zone of each pond with a D-shaped net (23 cm x 23 cm, 500 µm mesh size). The time effort of sampling was standardized to 10 minutes for each pond and the time of sampling within different mesohabitats (emergent, floating and submerged vegetation) was proportional to their relative abundance in each individual pond. Samples were fixed immediately on 70% ethanol. In the laboratory, samples were sieved over a mesh of 1 mm and all animals retained by the sieve were sorted, identified and counted using a stereomicroscope by using De Pauw and Vannevel [43]. Ephemeropterans, hemipterans and molluscs were identified to species level. Determination of dipterans was done up to family level. Other organisms were only sorted to higher taxomic level and counted (Acari and Hirudinaea to subclass, Trichoptera and Lepidoptera to order).

During August, we estimated the percentage of pond area covered by submerged, floating and emergent macrophytes and we inventoried the species composition of each of these vegetation types.

### 4: Data analysis

We used non-parametric Kruskall Wallis tests to test for an effect of pond management on total fish biomass and the relative biomass of dominant fish species. Furthermore, we applied parametric one-way ANOVA analyses and Tukey post-hoc tests to compare major pond characteristics (i.e. TN, TP, suspended matter, phytoplankton chlorophyll a, oxygen saturation, water transparency, cover by submerged and emergent vegetation, pond surface, water depth, thickness of the silt layer and pH) across different pond management types. For variables that were measured twice a year (spring and summer), we used averaged values from both sampling campaigns.

Taxon richness of each of the studied organism groups was used as measure of biological diversity. We defined ‘local richness’ as the average number of taxa recorded for an organism group in all ponds that belong to a specific management type. ‘Total richness’ was calculated as the total number of taxa found for a given management type. We based this calculation on a total of five randomly selected ponds per management type to standardize for pond number. We tested for differences in local richness among management types with parametric ANOVA and applied Tukey post-hoc tests to compare pairs of management types. Similarly, we analyzed the true Shannon index for local and total diversity (calculated as the exponent of the Shannon diversity indices) in order to also incorporate the evenness component of diversity in the evaluation of pond management [44].

We explored associations among the taxon richness (local and total) of organism groups using Principal Component Analyses (PCA) and we applied redundancy analysis (RDA) to formally test the overall effects of management. In addition, both for local and total richness, we applied Wilcoxon signed-rank tests to explore for consistent differences among pairs of management types across organism groups.

We defined ‘regional richness’ of an organism group as the total number of taxa recorded in a set of 20 randomly chosen ponds equally representing the five management types (4 ponds per management type). To evaluate the unique contribution of a management type to regional richness, we simulated the percentage change in regional richness that would result from the replacement of this management type by a random mixture of ponds belonging to the other management types. In order to assess the reproducibility of the result, this procedure was repeated 100 times for each pond management type per organism group.

National Red Lists of threatened species were used to count the number of rare plant [45] and hemipteran [46] species in each management type. These counts included species listed as ‘threatened’, ‘vulnerable’, ‘endangered’, ‘rare’ and ‘very rare‘. For zooplankton, we counted the number of nationally rare species as listed by Louette et al. [47].

With the exception of pH, all variables were logarithmically transformed prior to analysis. All univariate analyses were performed in STATISTICA 9.1 (StatSoft, Inc., Tulsa, Oklahoma). We used CANOCO 4.5 [48] for the multivariate analyses. The significance of the RDA models was evaluated with 999 random Monte Carlo permutations [49]. The simulations were written and performed in R [50]. One pond with NF management dried out unexpectedly during the summer of 2006 and was excluded from all analyses.

## Results

### 1: Fish community

**Figure 2:**
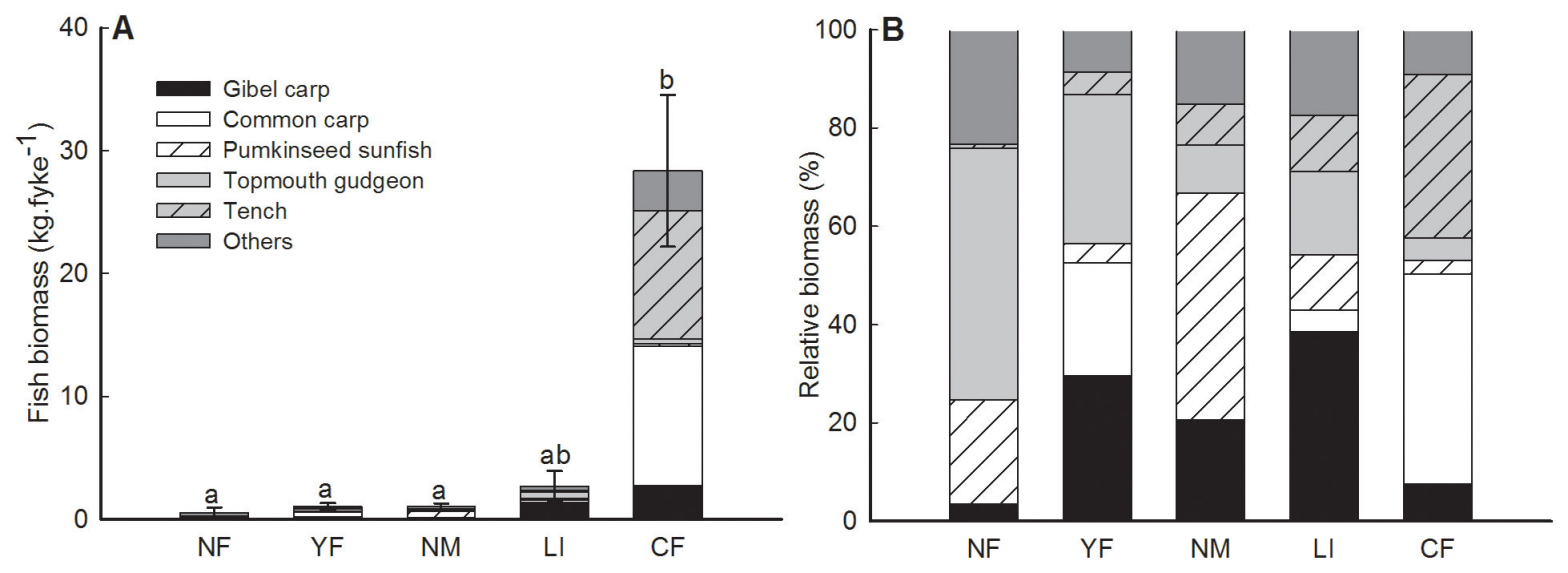
Bar plots showing the fish biomass (kg fyke^-1^) (A) and stacked bars with the relative biomass of most dominant fish species (B) for the different pond management types. Management types without letters in common (a, b) differ significantly in fish biomass (multiple comparison Kruskal-Wallis test, P<0.05). Error bars denote ± SE of the mean total fish biomass.

Pond management was strongly associated with total fish biomass and fish community composition (see Table S1). CF-ponds were characterized by a high total fish biomass (Figure 2 a) compared to the other management types and their communities were mainly dominated by common carp and tench (Figure 2 b). Fish communities in NF-ponds were dominated by topmouth gudgeon *Pseudorasbora* *parva* (Temminck and Schlegel), while NM-ponds contained more pumpkinseed sunfish *Lepomis* *gibbosus* (L.). YF-ponds were characterized by a relatively high abundance of small sized gibel carp *Carassius* *gibelio* (Bloch) and juvenile common carp. Despite the initial stocking of LI-ponds with rudd, roach, tench and pike, fish communities in these ponds tended to be dominated by gibel carp. Over the total set of ponds, piscivorous fish were not abundant. Only two ponds contained pike (one individual caught in a NM-pond and four individuals caught in a CF-pond) and over the total set of investigated ponds only small size classes of perch *Perca* *fluviatilis* (L.) (< 12 cm) were found. All management types were characterized by a strong proliferation of non-native fish species (60 to 80% of the total fish biomass, e.g. American brown bullhead *Ameiurus* *nebulosus* (Lesueur), common carp, gibel carp, pumpkinseed sunfish and topmouth gudgeon).

### 2: Pond characteristics

**Figure 3:**
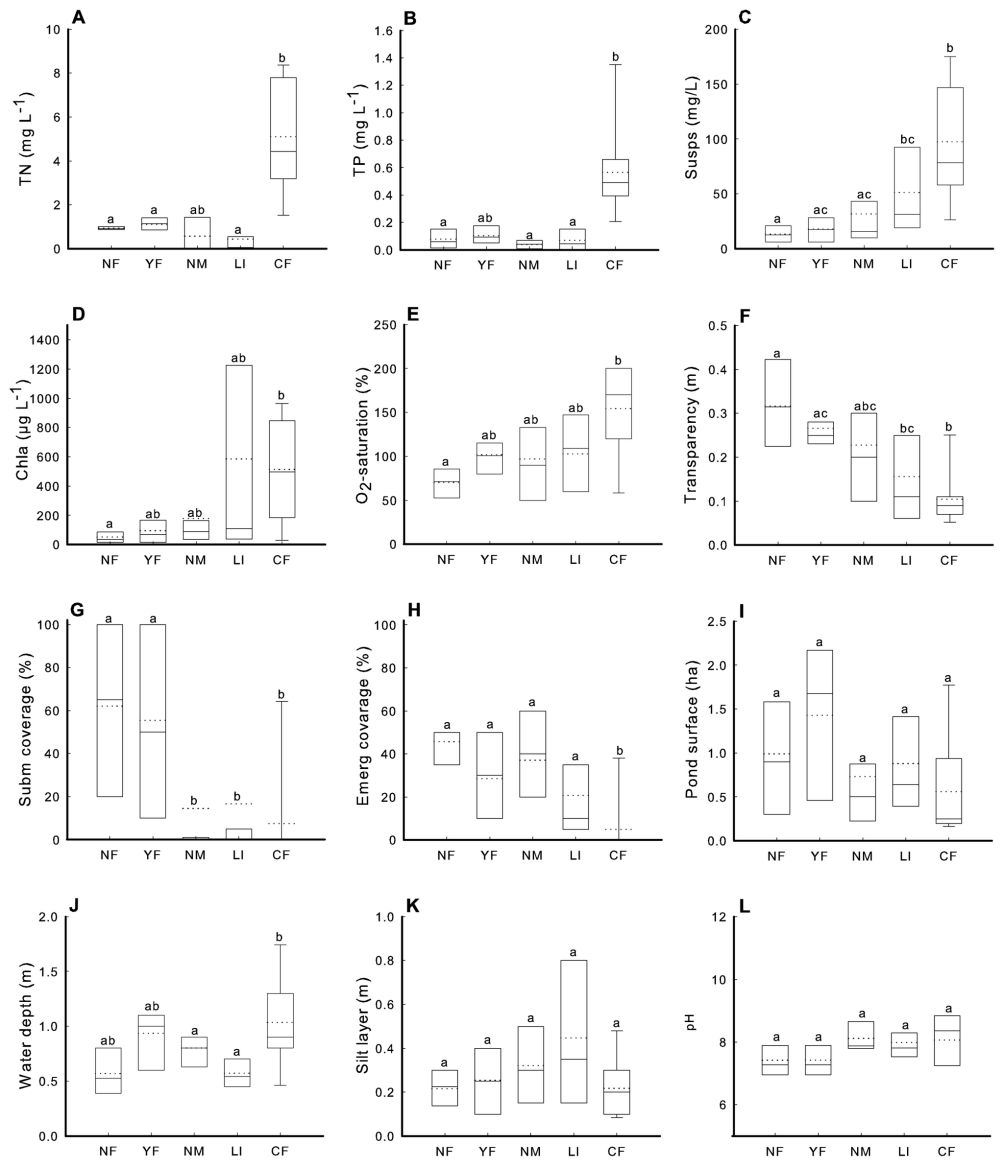
Box plots with the median (solid line) and the average (dotted line) of different pond characteristics in relation to pond management type. Boxes and error bars represent the 25th and 75th, and the 10th and 90th percentile respectively. (A) total nitrogen, (B) total phosphorus, (C) suspended solids, (D) chlorophyll *a*, (E) day time oxygen saturation, (F) water transparency, (G) percentage of coverage with submerged/floating vegetation, (H) percentage of coverage with emergent vegetation, (I) pond surface, (J) depth of water column, (K) thickness of the silt layer and (L) pH. Management types without letters in common (a, b, c) differ significantly from each other for the variable listed (Tukey HSD test, P<0.05).

Ponds exposed to different management types strongly differed with respect to a variety of important pond characteristics (Figure 3, see also Table S2). CF-ponds deviated most from other management types by a combination of very high TP and TN levels, high concentrations of suspended matter, chlorophyll *a* and daytime oxygen, low transparency and a low cover by submerged and emergent macrophytes (Figure 3). Conversely, NF and YF-ponds were typically characterized by well-developed submerged vegetation and high water transparency. Similar to CF-ponds, NM and LI-ponds were relatively turbid and contained no or only poorly developed submerged macrophyte vegetations, but tended to be more similar to NF and YF-ponds than to CF-ponds for nutrient concentrations and coverage by emergent vegetation. Pond size and thickness of the silt layer were not associated with pond management type (Figure 3). CF-ponds, however, tended to be deeper than NF and LI-ponds.

### 3: Local and total richness

**Figure 4:**
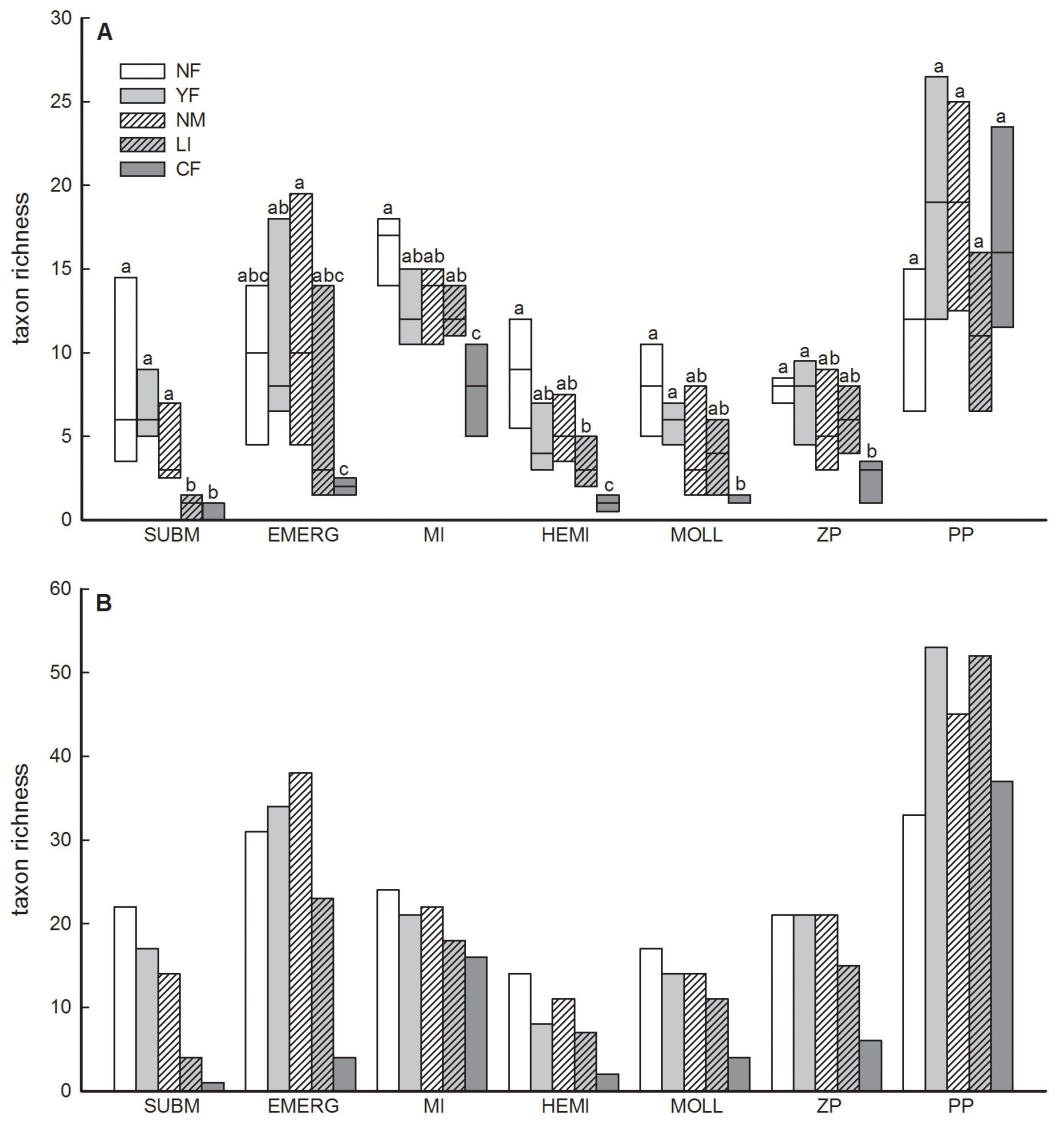
Box plots with the median local taxon richness (A) and bar plots of total taxon richness (B) for the studied organism groups in relation to pond management type. SUBM = submerged and floating vegetation, EMERG = emergent vegetation, MI = macro-invertebrates, HEMI = hemipterans, MOLL = molluscs, ZP = zooplankton and PP = phytoplankton. All groups are represented as species richness, except MI and PP where the number of families and number of genera are shown, respectively. Pond management types with average local richness values that do not differ significantly from each other (Tukey HSD test, P<0.05) are indicated by identical letters (a, b, c). Boxes represent the 25th and 75th percentiles.

With the exception of phytoplankton, management type significantly explained a large fraction of the variation in local richness of submerged and floating vegetation (63%), emergent vegetation (50%), aquatic macro-invertebrates (59%), hemipterans (70%), molluscs (45%) and zooplankton (48%) (Figure 4a; Table S3). Except for phytoplankton, local richness was consistently lowest in CF-ponds. The number of submerged macrophyte species was very low in LI-ponds (Figure 4 a). Tukey-tests performed for each organism group separately lacked power to reveal other differences among management types, but Wilcoxon signed rank tests on group means revealed consistent differences among management types across organism groups (Table S4). Local richness in NF, YF and NM-ponds proved to be consistently higher than in LI and CF-ponds. Similar patterns were observed for the differences in total richness of the different organism groups across management types (Figure 4 b, see also Table S4). Total richness indeed showed strong correlations with mean local richness (overall correlation: r = 0.85, p < 0.001; see also Figure S1), indicating a low impact of management type on beta diversity (data not shown). Very similar results were obtained for true Shannon diversity (Figure S2 and Table S5).

**Figure 5:**
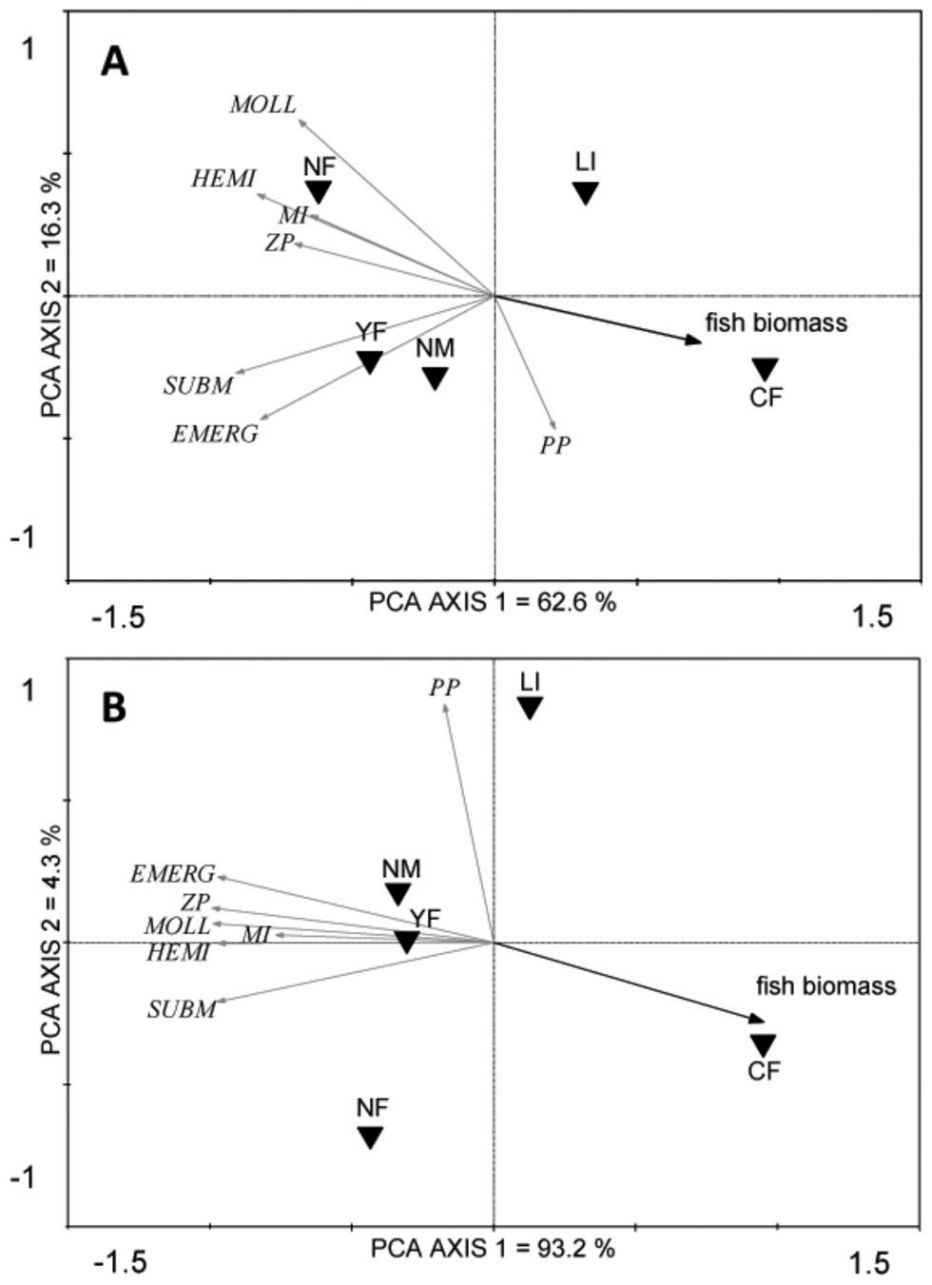
Biplot of a Principal Component Analysis (PCA) showing the associations of local taxon richness among groups and with fish biomass (A); and the associations of total taxon richness among groups with fish biomass (B). SUBM = submerged and floating vegetation, EMERG = emergent vegetation, MI = macro-invertebrates, HEMI = hemipterans, MOLL = molluscs, ZP = zooplankton and PP = phytoplankton. All groups are represented as species richness, except MI and PP where the number of families and number of genera are shown, respectively. Black triangles represent the centroids of the management types. The management centroids and fish biomass are plotted as supplementary variables to not influence the ordination.

With the exception of phytoplankton, both local and total richness tended to be positively associated among groups (Figure 5). RDA-analysis showed an overall strong negative association of the richness of most organism groups with fish biomass (local richness: R^2^ = 33.1 %, F = 11.402, p = 0.001; total richness: R^2^ = 85.0 %, F = 16.994, p = 0.028) (Figure 5).

### 4: Contribution of pond management types to regional richness

**Figure 6:**
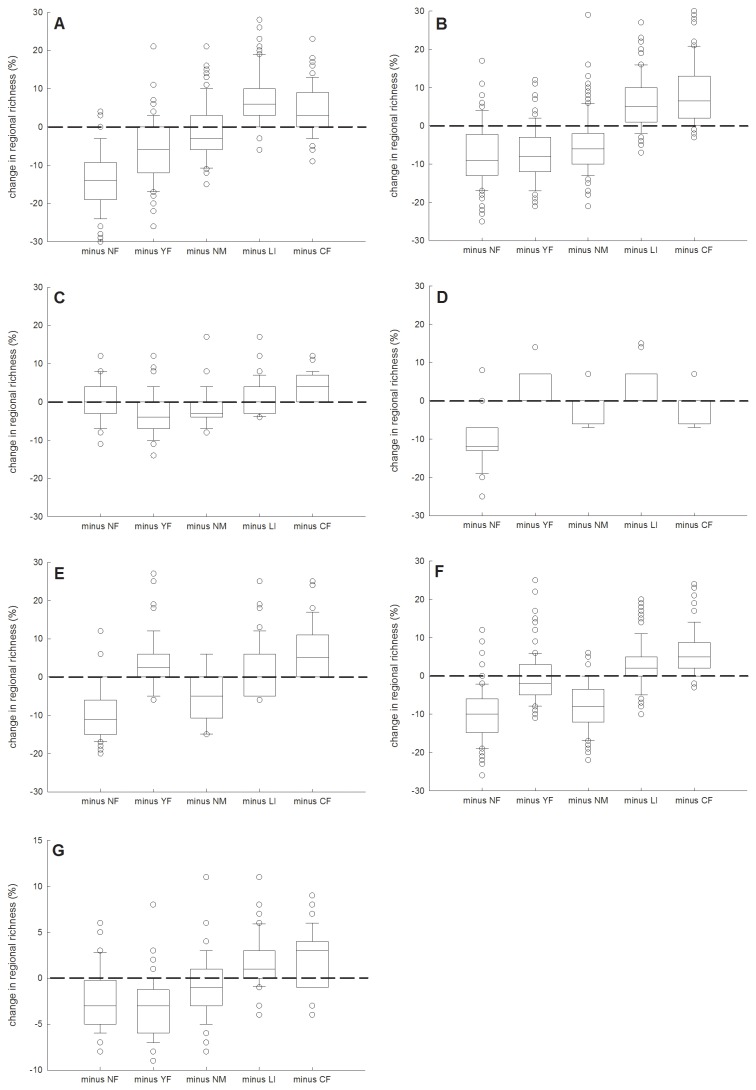
Boxplots with the median percentage change of regional taxon richness of the different organism groups upon replacement of management types by equally sized random mixtures of ponds belonging to other management types. (A) submerged and floating vegetation, (B) emergent vegetation, (C) macro-invertebrates, (D) hemipterans, (E) molluscs, (F) zooplankton and (G) phytoplankton. All groups are represented as species richness, except macro-invertebrates and phytoplankton where the number of families and number of genera are shown, respectively. Boxes and error bars represent the 25th and 75th, and the 10th and 90th percentile respectively. Open dots show outliers.

Pond management types differed strongly in the degree to which they contributed with unique species to regional richness (Table S6). For a hypothetical region consisting of 20 ponds with each management type being equally represented by 4 ponds, Figure 6 represents for each management type the estimated percentage of regional species loss or gain that would result from the replacement of this management type by a random selection of ponds of the other management types. Our simulations suggest that replacement of the NF management would result in a considerable reduction in the regional richness of most of the considered organism groups, especially submerged and emergent macrophytes, hemipterans, mollusks and zooplankton (Figure 6). Replacement of the YF management would mainly result in a reduction of the regional richness of submerged and emergent macrophytes, whereas replacement of the NM management would mainly lead to a loss of diversity in emergent macrophytes and zooplankton. CF and LI managed ponds contributed little to regional species richness. Conversely, for several organism groups replacement of these management practices would rather increase regional richness as a result of a stronger representation by other management types.

### 5: Occurrence of protected or rare species

Ponds exposed to different management types differed in the number of rare and threatened species they harbor (Figure S3). The highest number of protected species was consistently found in NF-ponds, and rare zooplankton species were only found in this management type. High numbers of rare and threatened plant species (submerged and emergent) were also found in YF-ponds. NM-ponds had relatively high numbers of threatened submerged plants. CF and LI-ponds contained no or only very low numbers of threatened species.

## Discussion

A profound understanding of the relation between management practices of ecosystems and biodiversity is crucial for the planning of effective nature conservation efforts [7,8]. Rather than being focused solely on the conservation of biodiversity in local communities, conservation ecology should be geared at maintaining biodiversity at larger spatial scale [51]. An understanding of the contribution of specific management practices to regional scale biodiversity is therefore pivotal. Given that the biodiversity responses of multiple organisms often tend to show low concordance [17,18,20], there is also a strong need for the simultaneous evaluation of management practices on local and regional diversity of multiple groups.

Our study shows that both the local and regional biodiversity of a broad variety of aquatic organism groups in a pond cluster are strongly determined by the way how ponds are managed. We also observed a strong concordance in the diversity response of the different organism groups to pond management, although the pattern observed for phytoplankton deviated from this general response. Management types differed in several aspects (fish stocking, biomass and composition of fish communities, frequency of pond drainage, nutrient addition through feeding), which complicated the interpretation of our results. Nevertheless, we found a strong negative association between overall community diversity in most organism groups and fish biomass. This is most clearly exemplified by the difference among the no fish (NF) and carp (CF) ponds. Although both types of ponds are drained annually during the winter, carp ponds were characterized by very high levels of nutrients, suspended matter and chlorophyll a, and by a low transparency and cover of submerged and emergent macrophytes. Local and total diversity of carp ponds were consistently low in all organism groups (except for phytoplankton) and contributed with almost no unique taxa to the regional diversity of the area. The poor ecological quality and low diversity of carp ponds is probably the result of a variety of mechanisms that act simultaneously. Through benthic foraging, large size classes of common carp and tench resuspend sediments [52,53] and algae [54], and enhance water turbidity and internal eutrophication [55,56], which ultimately result in the loss of submerged and emergent macrophytes. The concomitant loss of habitat structure and refuges combined with high predation pressure by the fish are most probably responsible for the loss of invertebrate diversity [28,57,58]. Such effects are enhanced if fish farmers add artificial food, strengthening eutrophication and associated phytoplankton blooms.

A management that was directed at maximally preventing the establishment of fish populations in the ponds (NF) yielded clear water with well-developed aquatic vegetation, with high levels of local and total richness in almost all of the investigated organism groups. NF-ponds also contained relatively high numbers of rare and protected species and our simulations suggest that omission of this management type in the area would result in a substantial reduction of the regional richness of a variety of organism groups, such as macrophytes, hemipterans, mollusks and zooplankton. The high conservation value of NF-ponds probably resulted from a combination of very low fish densities with annual drainage during winter. Periodic pond drainage can stimulate the development of submerged vegetation [59,60] and strengthen the mechanisms that stabilize the clear water macrophyte dominated state, especially in systems that are subjected to eutrophication (cf. theory of alternative stable states [28,58,61,62]:). Through increased habitat complexity, food availability and shelter for prey against predation [28,61,63,64] the development of macrophyte vegetations can contribute to a higher biodiversity in other groups of aquatic biota. In addition, regular drainage stimulates the decomposition of organic matter in the sediments and promotes the establishment of typical pioneering Littoretea vegetation of which many members are considered of high conservation concern in Western Europe [eg. *Elatine* *triandra* (Schkuhr), *Baldellia* *repens* (Lam.), *Apium* *inundatum* (L.)] [65,66]. Although CF-ponds are also drained annually, these ponds lacked high levels of diversity. The positive effects of drainage in these ponds are seemingly offset by their high fish biomass.

Ponds in use for the commercial farming of young-of-the-year fish (YF) were characterized by extensive macrophyte stands and a high water transparency, and contributed disproportionally to the regional richness of emergent and submerged macrophytes. Biodiversity levels for most groups in YF-ponds were consistently high. As in NF-ponds, the high ecological quality and biodiversity of YF-ponds is likely the result of a combination of annual drainage during winter and stocking of low biomasses of fish in late spring.

In the absence of any management (NM), ponds contained no or only a sparse vegetation of submerged macrophytes. The local and total diversity of most organism groups were relatively high. The absence of management is a cheap option that seems to be associated with reasonable levels of biodiversity in most groups. Despite its low short term costs, we would advise against this option for several reasons. First, the absence of submerged macrophyte vegetation is striking and is probably related to the permanency of high water levels. Fish were probably not responsible for the absence of submerged macrophytes, given the relatively low total fish biomass and dominance by small-bodied species. The absence of macrophytes results in poor habitat heterogeneity and we suspect that this would lead to an impoverishment of most of the invertebrate and zooplankton fauna on a longer term [61,64]. Second, we expect that the absence of periodic drainage would inevitably result in the loss of the ponds due to succession and filling. We therefore do not consider zero management as a valid management option for the longer term.

Surprisingly, the ecological quality of ponds with low intensity management (LI) proved to be low. Limnological characteristics were similar for LI-ponds and ponds without management (NM), and submerged vegetation was nearly absent in both types of ponds. In addition, a considerable fraction of LI-ponds contained very high levels of suspended matter and phytoplankton chlorophyll a. Levels of taxon richness were systematically lower in the LI than in the NF, YF and NM-ponds. Most probably, this poor quality was the result of a combination of low drainage frequency combined with a high biomass of relatively large-bodied benthic fish, mostly gibel carp. The observed fish communities failed to reflect the composition of the initially stocked fish (i.e. rudd, roach, tench and pike), and tended to be higher in fish biomass than the NF and YF-ponds, probably as the result of inadequate drainage. In contrast to the NF and YF-ponds, where drainage was carefully performed and where considerable efforts were done to keep out fish from nearby rivulets, the winter drainage of LI-ponds is often carried out with less care. Some species, like gibel carp, are robust and can survive extended periods in harsh conditions in small puddles and pools [67]. Careful drainage may therefore be a prerequisite for any management type aiming at sustaining pond biodiversity.

## Conclusion

We observed a remarkable concordance in the diversity response of organism groups to pond management types in the studied pond cluster. A management focusing on keeping ponds free of fish via repeated and carefully applied pond drainage combined with additional measures aimed at preventing fish from entering ponds through inlets appeared to be the best guarantee for high local diversity across organism groups. Such management also resulted in the strongest contribution to regional biodiversity and supported high numbers of rare and endangered species. Based on these results we conclude that such management forms an important tool for the maintenance of aquatic biodiversity in ponds and should therefore be regarded as an essential part of regional conservation plans for pond areas, at least in eutrophied regions like Flanders where fish populations often spontaneously develop to high densities. Alternative management types, such as the commercial farming of juvenile cyprinids, also resulted in good ecological quality and high levels of biodiversity, presumably due to the combination of regular periodic drainage and stocking of only a low fish biomass relatively late in the season, allowing for the development of aquatic vegetation. Although zero management is by far the cheapest non-commercial option on the short term, we do not recommend this management type because it does not support the development of submerged macrophytes vegetations. Furthermore, this management involves a serious risk of losing valuable ponds on a longer time scale due to succession and filling. Zero or low-intensity pond management is often applied by nature conservation organizations in Europe because of lack of financial resources, but involves a risk of gradual deterioration of ponds on a longer term. When strictly regulated, specific types of commercial fish farming have considerable potential to contribute to the conservation of regional nature values in semi-natural meso- to eutrophic pond clusters, especially when such activities combine regular pond drainage with low fish stock biomass and when the use of fertilizers and artificial feeds are avoided.

## Supporting Information

Figure S1 Association between mean local and total richness across management types for all investigated biota together (r = 0.85, p <0.001) (a) and for organism groups separately; SUBM = submerged and floating vegetation (r = 0.99, p = 0.002) (b), EMERG = emergent vegetation (r = 0.99, p = 0.001) (c), MI = all taxa of macro-invertebrates (r = 0.91, p = 0.030) (d), HEMI = hemipterans (r = 0.96, p = 0.008) (e), MOLL = molluscs (r = 0.95, p = 0.013) (f), ZP = zooplankton (r = 0.92, p = 0.027) (g), PP = phytoplankton (r = 0.28, p = 0.65) (h).
(TIF)

Figure S2 **Box plots with the median local true Shannon diversity (a) and bar plots of total true Shannon diversity (b) for the studied organism groups in relation to pond management type.**
SUBM = submerged and floating vegetation, EMERG = emergent vegetation, MI = macro-invertebrates, HEMI = hemipterans, MOLL = molluscs, ZP = zooplankton and PP = phytoplankton. All groups are presented at the species level, except MI and PP where the number of families and number of genera are shown, respectively. Pond management types with average local diversity values that do not differ significantly from each other (Tukey HSD test, P<0.05) are indicated by identical letters (a, b, c). Boxes represent the 25th and 75th percentile.
(TIF)

Figure S3 **The number of protected species of submerged/floating plants (SUBM), emergent plants (EMERG), aquatic macro-invertebrates (without Diptera and Molluscs), and the number of national rare zooplankton species (ZP) observed in each pond management type.**
(TIF)

Table S1 **Results of Kruskal-Wallis analyses testing for effects of pond management type on total fish biomass and the relative biomass of the dominant fish species.**
Df = degrees of freedom, H = H-values, P = p-value.
(XLSX)

Table S2 **Results of one-way ANOVA analyses testing for effects of pond management type on pond characteristics.**
Df = degrees of freedom, SS = Sum of Squares, MS = Mean Squares, F = F-ratio, P = p-values.
(XLSX)

Table S3 **Results of ANOVA-analyses testing for effects of pond management type on the local richness of each of the studied organism groups.**
All groups are represented as species richness, except MI and PP where the number of families and number of genera are shown, respectively. Df = degrees of freedom, SS = Sum of Squares, MS = Mean Squares, F = F-ratio, P = p-values.
(XLSX)

Table S4 **Results of Wilcoxon signed-rank tests testing for differences in local richness and total richness among pairs of pond management types across organism groups.**
(XLSX)

Table S5 **Results of ANOVA-analyses testing for effects of pond management type on the true local diversity of each of the studied organism groups.**
Df = degrees of freedom, SS = Sum of Squares, MS = Mean Squares, F = F-ratio, P = p-values.
(XLSX)

Table S6 **List of taxa uniquely found to be present in each of the management types during the course of the study.**
(XLSX)
