# Supplementary figures and images for: How to Maximally Support Local and Regional Biodiversity in Applied Conservation? Insights from Pond Management
Source: PLoS One. 2013 Aug 12;8(8):e72538. doi: 10.1371/journal.pone.0072538 (PMC3741229; doi:10.1371/journal.pone.0072538)

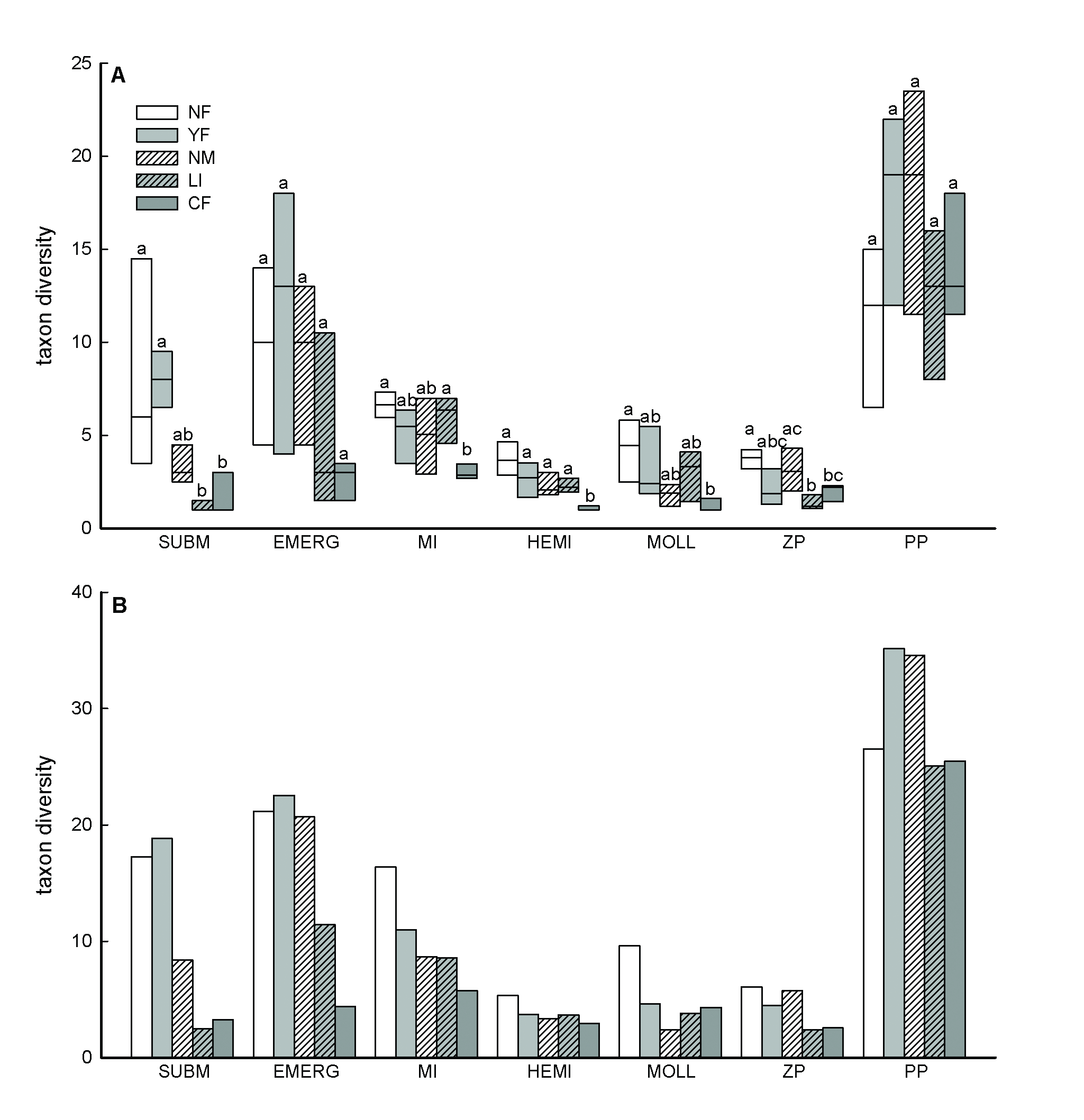

Supplement: Figure S2 — Box plots with the median local true Shannon diversity (a) and bar plots of total true Shannon diversity (b) for the studied organism groups in relation to pond management type. SUBM = submerged and floating vegetation, EMERG = emergent vegetation, MI = macro-invertebrates, HEMI = hemipterans, MOLL = molluscs, ZP = zooplankton and PP = phytoplankton. All groups are presented at the species level, except MI and PP where the number of families and number of genera are shown, respectively. Pond management types with average local diversity values that do not differ significantly from each other (Tukey HSD test, P<0.05) are indicated by identical letters (a, b, c). Boxes represent the 25th and 75th percentile. (TIF) [file pone.0072538.s002.tif]

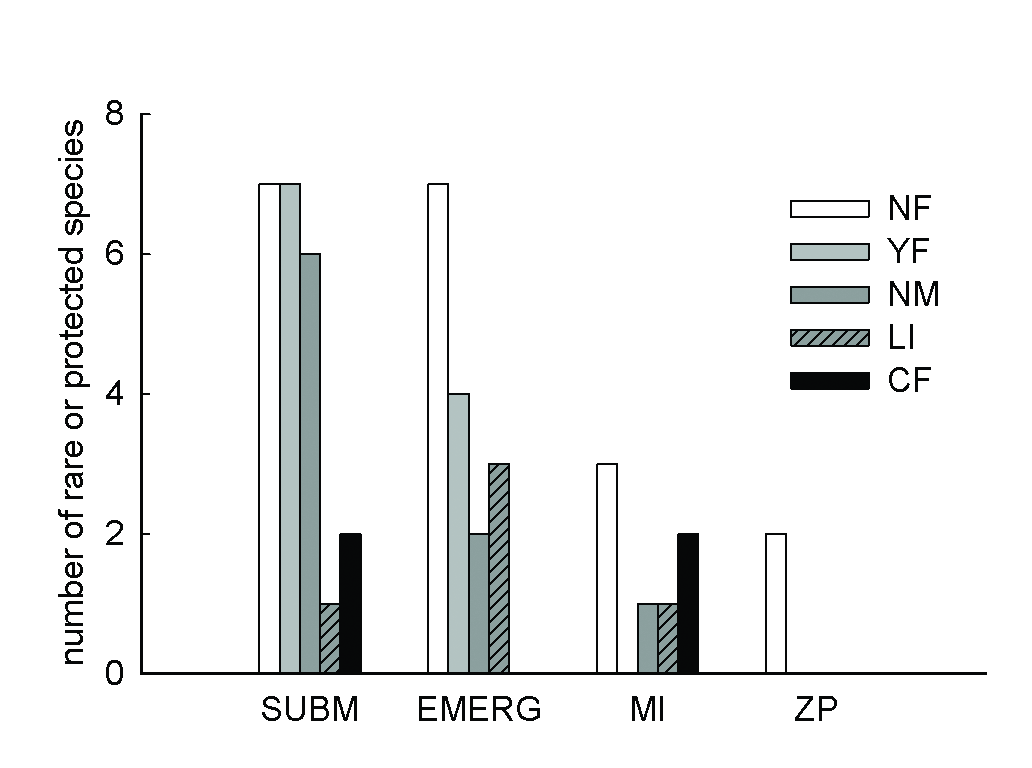

Supplement: Figure S3 — The number of protected species of submerged/floating plants (SUBM), emergent plants (EMERG), aquatic macro-invertebrates (without Diptera and Molluscs), and the number of national rare zooplankton species (ZP) observed in each pond management type. (TIF) [file pone.0072538.s003.tif]
